# Technical evaluation of language models adapted for the automation of legal contracts: clause extraction, classification, and summarization

**DOI:** 10.3389/frai.2026.1782405

**Published:** 2026-03-26

**Authors:** Jaime Govea, Iván Ortiz-Gárces, Pablo Palacios, Alexandra Maldonado Navarro, Santiago Acurio Del Pino, William Villegas-Ch

**Affiliations:** 1 Escuela de Ingeniería en Ciberseguridad, Facultad de Ingeniería y Ciencias Aplicadas, Universidad de Las Américas, Quito, Ecuador; 2 Escuela de Informática y Telecomunicaciones, Universidad Diego Portales, Santiago, Chile; 3 Escuela de Posgrados, Maestría en Derecho Digital, Universidad de Las Américas, Quito, Ecuador; 4 Facultad de Derecho y Sociedad, Pontificia Universidad Católica del Ecuador, Quito, Ecuador

**Keywords:** artificial intelligence, clause extraction and summarization, domain-adapted language models, legal contract automation, legal NLP evaluation metrics

## Abstract

The growing demand for automation in legal contract management exposes a persistent limitation of current language models: insufficient adaptation to the semantic, structural, and regulatory constraints of legal language. While large language models perform well on general NLP tasks, their direct application to legal document classification, clause extraction, and contract summarization often yields unstable, legally unreliable outputs. This work presents a structured methodological pipeline for evaluating and adapting language models for legal contract automation, combining domain-specific fine-tuning of open-source models with a controlled comparative assessment against large general-purpose LLMs used exclusively in inference mode. The methodology integrates legal corpus curation, clause-level annotation, and efficient adaptation techniques, and is evaluated across three core tasks: contract document classification, normative clause extraction, and regulatory summarization. The evaluation protocol is explicitly designed to disentangle the effects of supervision from deployment constraints arising in regulated legal settings. Experimental results show consistent and statistically significant performance gains for legally adapted models over general-purpose baselines, achieving Macro-F1 of 0.921 in classification, span-level F1 of 0.903 in clause extraction, and ROUGE-L of 0.886 in summarization (*p* < 0.01). Robustness analysis and cross-validation confirm stability across heterogeneous private-sector contract types. The findings should be interpreted under the evaluated comparison regime and highlight that, in legally constrained multi-stage workflows, task-aligned supervision provides measurable structural benefits that are not reducible to model scale alone when general-purpose LLMs are restricted to inference-only deployment.

## 1 Introduction

The growing adoption of large language models (LLMs) has transformed multiple areas of natural language processing (NLP), including automatic text generation, semantic analysis, and complex question answering (Herwanto et al., 2024). Despite these advances, their application in regulatory and legal environments remains a challenging problem due to the highly structured, context-dependent, and formally constrained nature of legal language. Contracts, regulations, and administrative acts are characterized by dense normative references, jurisdiction-dependent interpretations, and specialized semantic conventions, where minor textual deviations may lead to significant legal consequences. Under these conditions, the direct transfer of models pre-trained on general-purpose corpora to legal tasks often yields unstable or non-interpretable outputs, thereby limiting their practical reliability (Thakur et al., 2023).

This work addresses this limitation by designing and evaluating a structured, task-oriented adaptation and evaluation pipeline to automate contractual processes in the private sector. This work addresses this limitation by designing and evaluating a structured, task-oriented adaptation and evaluation pipeline to automate contractual processes in the private sector. Rather than introducing a new language model architecture, the study focuses on formalizing a controlled experimental regime that disentangles the effects of supervision, deployment constraints, and domain alignment within legally constrained workflows. Specifically, it combines domain-specific fine-tuning of open models with a controlled comparative evaluation against large general-purpose LLMs operating exclusively in inference mode, thereby characterizing performance under realistic access and supervision conditions. The proposed pipeline targets routine but high-impact contractual operations, such as document classification, clause identification, and regulatory summarization, which are central to contract review and update processes in dynamic legal environments (Latif and Zhai, 2024).

Unlike prior studies that focus exclusively on isolated legal classification tasks (Chalkidis et al., 2020; Lobanchykova et al., 2021) or on language model-assisted document comparison and reformulation (Narendra et al., 2024), this work adopts an integrated processing pipeline perspective. The proposed structured pipeline encompasses identifying relevant contractual clauses through hierarchical classification, assessing inter-clause semantic consistency, and generating context-aware summaries aligned with regulatory constraints. These components are evaluated using segmented, anonymized, and standardized real-world contracts, enabling the analysis of functional behavior under conditions representative of private-sector legal practice. Methodologically, the pipeline incorporates a hybrid training strategy that combines curated legal data with controlled synthetic augmentation to enhance semantic coverage without compromising legal validity. Stratified cross-validation and Wilcoxon statistical tests are employed to ensure that observed performance differences are not attributable to random variation (Sills, 2024).

The evaluation is conducted along three complementary dimensions: classification accuracy, clause-level extraction quality, and robustness of generated summaries under heterogeneous contractual scenarios. Performance is assessed using established metrics, including Macro-F1, span-level F1, and ROUGE-L, supported by variability analysis and confidence intervals. The evaluation indicates that performance differences are regime-dependent: domain-adapted models exhibit greater structural stability and span-level consistency under supervised alignment, whereas large-scale LLMs demonstrate comparative strengths primarily in inference-driven generative flexibility. These findings are interpreted relative to the evaluated deployment and supervision configurations rather than as absolute architectural claims.

The contributions of this work are articulated at three levels. From a technical perspective, it demonstrates that effective domain adaptation and structured evaluation strategies can yield reliable performance in regulated legal tasks without requiring prohibitive computational resources. From a methodological standpoint, it introduces a reproducible, task-oriented evaluation-and-adaptation pipeline that explicitly characterizes supervision-versus-scale trade-offs under realistic deployment constraints, enabling a more rigorous interpretation of domain adaptation effects in legally constrained NLP settings. From a functional perspective, it provides empirical evidence supporting the feasibility of automating contract review and update processes while preserving legal consistency within auditable and structured workflows.

This work does not aim to replace human legal expertise but to support legal professionals through auditable, reproducible, and context-aware analytical tools. By positioning language models as decision-support components rather than autonomous legal agents, the proposed structured pipeline contributes to the responsible integration of AI into legal document management, reinforcing legal certainty while improving operational efficiency (Wei et al., 2024).

## 2 Literature review

The use of LLM models in the legal and administrative fields has led to significant growth in research focused on their adaptation to specific domains, utilizing fine-tuning techniques, and additional pre-training. This work has addressed tasks such as legal document classification, contractual clause extraction, regulatory summaries generation, and named entity recognition (NER) (Li et al., 2024). Recent literature indicates a clear trend toward models specifically trained on legal corpora outperforming generalist models, not only due to their quantitative advantages but also due to their linguistic and structural adaptation to legal language.

Recent surveys on large language models in the legal domain further confirm this trend. Siino et al. (2025) provide a systematic review of current legal NLP approaches based on LLMs, covering applications such as legal reasoning, contract analysis, statutory interpretation, and case law retrieval. Their analysis highlights the rapid expansion of LLM-based systems in law while identifying persistent challenges in reproducibility, domain adaptation, and structured evaluation across heterogeneous legal tasks. Notably, the survey emphasizes that many existing works evaluate isolated tasks without integrating multiple legal processing stages into coherent analytical pipelines. This gap motivates the need for structured, task-oriented evaluation and adaptation pipelines such as the one developed in the present work.

One of the fundamental advances in legal document classification is the study by Zheng et al. (2021), which introduces the CaseHOLD dataset—a collection of over 53,000 examples designed to identify legal precedents. This work demonstrates that specific pretraining on legal corpora yields substantial performance improvements over models such as BERT, which are trained on generic data, with a 7.2% increase in F1. This finding has been supported by Chalkidis et al. (2020), who developed a family of models called Legal-BERT, specifically pretrained on legal texts. Their multitasking evaluation of the LexGLUE benchmark confirmed that domain tuning not only improves accuracy but also stability in tasks such as multilabel document classification (EURLEX) and sentence prediction (CaseHold). Additional evidence on the importance of domain and task adaptation in legal NLP is provided by Tagarelli and Simeri (2021); Simeri et al. (2023), who explore unsupervised law article mining and LamBERTa-based adaptation strategies for article retrieval in the Italian Civil Code. Their findings reinforce the idea that structurally specialized pretraining and task-specific adaptation significantly improve retrieval and reasoning performance in codified legal systems, further supporting the need for domain-aware modeling strategies.

In contract clause extraction, the LEDGAR corpus served as an empirical benchmark for comparing generalist models with adapted models. In the analysis by Jayakumar et al. (2023), it showed that generalist models such as LLaMA-2 or Falcon-180b, although competent in general tasks, lose up to 26.8 % of F1 in the thematic classification of contractual clauses compared to smaller optimized legal models, demonstrating that size does not compensate for the lack of semantic alignment with the domain.

Regarding the generation of regulatory or legislative summaries, the retrieval-augmented generation (RAG) approach has proven effective in contextual alignment tasks in legal texts. This model has been validated in recent studies, such as that by Vats et al. (2023), which showed a significant improvement in the quality of legal summaries generated by integrating pre-generator retrieval systems and AI-based legal reasoning models.

NER in legal contexts has significantly advanced with few-shot learning techniques. FsPONER, a recent proposal, was shown to outperform conventionally tuned models by 10 % in F1 when using TF-IDF vectors as the base semantic representation for data-scarce scenarios (Tang et al., 2024). Furthermore, a comparative analysis performed on eleven multilingual LLMs revealed significantly variable performance between proprietary and open models, highlighting the capabilities of models such as GPT-4, although with reservations regarding its lack of interpretability and dependence on closed resources.

Beyond technical performance, several authors have highlighted the importance of considering ethical aspects when fine-tuning and using LLMs for legal tasks. Tripathi et al. (2024), emphasize the risks of racial bias in fine-tuned legal models, suggesting that even in highly regulated environments, LLMs can reproduce discriminatory associations if the base corpus is not adequately filtered. Malic et al. (2023) address ethnic and gender bias in fine-tuned models, highlighting the potential harm in sensitive legal contexts. These findings have motivated proposals, such as active data curation (Bender et al., 2021) and the use of semantic intervention techniques, like CSAFT (Li et al., 2024), which can generate more diverse synthetic samples to strengthen fit and mitigate bias.

Regarding privacy and security, Yao et al. (2024) present a comprehensive analysis of the risks inherent to LLMs, including sensitive data leakage, inadvertent exposure of confidential information, and parameter extraction vulnerabilities. Their work highlights the need for regulatory compliance, especially in contexts such as Europe, where the GDPR imposes specific data protection obligations. In addition, Pesch (2025) analyzes the challenges faced by AI systems in administrative processes, highlighting the importance of ensuring algorithmic transparency and accountability.

The reviewed literature converges on the importance of adapting language models to the structural, semantic, and normative characteristics of legal corpora, consistently reporting performance gains under domain-specific fine-tuning. However, most prior studies evaluate domain adaptation effects in isolation, without explicitly distinguishing between supervision regimes, deployment constraints, and access conditions that characterize real-world legal environments. In particular, comparisons between adapted legal models and large general-purpose LLMs often do not clarify whether observed differences arise from domain specialization, supervised alignment, model scale, or asymmetric fine-tuning accessibility. Consequently, while the effectiveness of legal-domain adaptation is well documented, the literature provides limited insight into how supervision-versus-scale trade-offs behave under realistic deployment regimes. This gap motivates the present work, which formalizes and evaluates a controlled comparison protocol that characterizes these interactions within structured, multi-stage legal automation workflows.

## 3 Materials and methods

### 3.1 Problem definition and experimental scope

The processing of administrative legal documents represents a complex challenge for general-purpose language models due to the structural, terminological, and syntactical peculiarities that characterize this type of text. Unlike the language used in open domains, such as social media or news, administrative and regulatory documents are governed by hierarchical discursive logic, with intensive use of technical terms, conditional clauses, and explicit legal references. This formal structure requires NLP models not only to understand the semantics of the content but also to interpret normative relationships, identify legal entities, and operate with a high level of contextual accuracy. Under these conditions, language models pre-trained on large generic corpora tend to present significant limitations in specialized tasks, given that their knowledge is widely but superficially distributed concerning specific domains.

The need to adapt language models to specialized contexts has driven the development of fine-tuning techniques and specific pre-training on corpora aligned with the application domain. In the case of legal and administrative language, this adaptation is critical to ensure robust performance in fundamental tasks such as the classification of regulatory documents, the automated extraction of contractual clauses, and the generation of coherent and legally valid summaries (Bozdag et al., 2024). These tasks, in addition to requiring a deep semantic understanding, necessitate that the model recognize recurring discourse patterns and regulatory elements, such as articles, provisions, annexes, and cross-references. In practice, these functions are essential for reducing the manual workload in document review processes, bidding processes, audits, regulatory compliance analysis, and contract management.

This paper addresses three leading tasks representative of legal and administrative processing: document classification, clause extraction, and the generation of regulatory summaries. Document classification consists of assigning thematic or functional categories to regulatory or administrative texts, such as resolutions, contracts, general provisions, or administrative procedures. This task is essential for the efficient organization and retrieval of information in legal systems and institutional databases. The second task, clause extraction, involves identifying specific text segments that contain critical information, such as contractual terms, obligations, terms of validity, warranties, or dispute resolution mechanisms. This extraction must be accurate, as any error can alter the legal interpretation of the text. Ultimately, the goal of generating regulatory summaries is to produce concise versions of legal documents, preserving their informational validity and logical structure. This is especially useful in scenarios where large volumes of information must be reviewed in a short timeframe (Draper and Gillibrand, 2023).

The experimental scope focuses on a controlled comparative evaluation under distinct supervision and deployment regimes. Rather than conducting a purely architectural comparison, the objective is to characterize how performance behaves when open-weight models are subjected to supervised domain adaptation while large general-purpose LLMs are accessed under inference-only constraints (Viegas et al., 2023). This distinction reflects realistic deployment asymmetries commonly encountered in regulated environments, where proprietary foundation models are typically accessible only via API-based inference, whereas open models permit domain-specific fine-tuning.

Accordingly, models such as BERT and T5 in their base versions are used as representatives of generalist architectures, and their performance is compared with legally adapted variants, including Legal-BERT, as well as open models fine-tuned using efficient techniques such as Low-Rank Adaptation (LoRA) and Quantized LoRA (QLoRA). The comparison is conducted using standardized, publicly accessible legal datasets selected for their relevance, accurate annotation, and representativeness of legal and administrative language. This experimental framing allows the study to isolate the interaction between supervision, domain alignment, and deployment constraints, rather than attributing differences solely to model scale or architectural specialization.

### 3.2 Data sets used

To evaluate the performance of language models in specialized legal tasks, three datasets widely recognized in the literature for their relevance, annotation quality, and topic coverage were selected. These datasets complement each other in covering the tasks of document classification, contract clause extraction, and regulatory summary generation, and represent different levels of granularity and discursive complexity. Each of them has been preprocessed and adapted to the input format required by the evaluated models, respecting their original legal structure.

The first corpus used corresponds to the LexGLUE benchmark, a collection of tasks focused on understanding legal language in English, developed to evaluate models in legal environments using standardized metrics (Chalkidis et al., 2022). Two subcomponents of the benchmark are used in this work: EURLEX and CaseHold (Zheng et al., 2021; Prajwal and Kumar, 2022). The EURLEX dataset comprises over 55,000 legislative documents retrieved from the official EUR-Lex portal of the European Union, each labeled with multiple subject codes representing legal concepts defined by the EuroVoc classification. The task is multilabel, since a single document can be associated with multiple subject domains. CaseHold, for its part, contains 53,000 pairs of excerpts from court rulings in a multiple-choice question-and-answer format, where the correct decision must be identified among five options. This task involves complex legal inference and requires structural reasoning skills and knowledge of jurisprudence.

The second dataset used is LEDGAR, specifically designed for the classification of contractual clauses (Tuggener et al., 2020). This corpus comprises approximately 850,000 clauses extracted from public legal contracts, primarily corporate contracts, and is classified into more than 100 functional categories. Each entry represents a stand-alone clause, segmented from the original contractual body and manually annotated according to its normative content. Unlike other contract corpora, LEDGAR focuses on the diversity of syntactic structures, making it a suitable resource for evaluating the models' ability to recognize discursive patterns in fragmented and legally dense texts.

The third set is the EURLEX Summarization Corpus, derived from European legislative documents accompanied by official summaries written by legal experts (Aumiller et al., 2022). This corpus includes full texts in various official languages of the European Union, although for this study, only the English version was used. Each document-summary pair constitutes an instance for training and evaluating text generation models. Unlike other collections of generic summaries, this corpus maintains explicit semantic alignment with the structural elements of the source document, such as titles, regulatory sections, and annexes, which enables the evaluation of both the coherence and thematic coverage of the generated summary.

Before being incorporated into the models, each dataset undergoes a structured normalization and preparation process. Initial text processing involves three fundamental operations: legal tokenization, structural cleaning, and thematic filtering (Schmidt and González, 2020). Since legal documents contain abundant formal markers, cross-references, and normative enumerations, a semantic tokenizer based on the WordPiece scheme, adapted to legal vocabularies, has been implemented (Song et al., 2021). Formally, for a document d∈D, a tokenization function $T:D\to {\mathbb{R}}^{n\times k}$ is applied, where each *d* is represented as a sequence of *n* tokens embedded in a *k*-dimensional vector space, as defined in Equation (1)


$$\begin{array}{l} T\left(d\right)=\left[{\text{t}}_{1},{\text{t}}_{2},\ldots ,{\text{t}}_{n}\right],\;{\text{t}}_{i}\in {\mathbb{R}}^{k} \end{array}$$ (1)

Subsequently, a normalization stage based on legal rules is applied, in which elements that cannot be interpreted by the models, such as redundant headings, institutional seals, and editorial notes, are eliminated without compromising the normative integrity of the document. Finally, thematic filtering consists of selecting only those instances that present at least one valid and verifiable annotation according to the established categories, represented as labels *y*∈{0, 1}^*m*^, where *m* is the total number of legal classes or clauses considered.

Preprocessing ensures that the models operate on homogeneous, coherent inputs aligned with formal legal structures. Furthermore, the balanced proportions of classes were verified to avoid bias in the training phase, and a subset of the data was reserved for cross-evaluation in each of the tasks considered.

### 3.3 Language models evaluated

Comparative performance evaluation in legal and administrative tasks requires the inclusion of models of different architectural natures and levels of semantic specialization. This study considered both general reference models widely used in NLP and models specifically designed or adapted for the legal domain. This diversity allows us to precisely identify the structural and functional advantages offered by model specialization compared to general configurations and to quantify the impact of fine-tuning on specific tasks.

#### 3.3.1 General-purpose models

Among the selected general-purpose models is BERT-base, proposed by Devlin et al. (2019), which constitutes a bidirectional transformer architecture trained on a generic corpus (BooksCorpus and Wikipedia). This model has been widely used as a starting point for classification, information extraction, and semantic analysis tasks due to its ability to represent contextual relationships of tokens through bidirectional attention. BERT was used in this study as the reference model for document classification and clause extraction tasks, utilizing its base version with 110 million parameters and a 12-layer structure, comprising 12 attention heads and an embedding dimension of 768.

For the automatic summarization task, the T5-base model was selected, which adopts an encoder-decoder architecture trained with a unified task formulation under the “text-to-text” paradigm (de Jong et al., 2023). T5 was pre-trained on the C4 corpus and has proven effective in structured generation tasks due to its multi-task transfer-oriented design. In this work, the base version with 220 million parameters was fine-tuned and prompt-tuned for normative summary generation on the EURLEX corpus.

In unsupervised inference scenarios, variants of GPT-3.5 and GPT-4 were also considered, operating under zero-shot and few-shot inference settings. These one-way attention-based autoregressive models were used without parameter tuning, relying solely on explicit instruction (prompt engineering), which enables their generalization capacity to be evaluated without additional training in the legal domain. Their use is justified by the interest in determining whether the model's massive scale compensates for its lack of specialization, particularly in legal generation and reasoning tasks such as CaseHold.

#### 3.3.2 Legal domain-specific models

The models adapted to the legal domain have been selected based on their thematic coverage, compatible architectural structure, and public availability. The first of these is Legal-BERT, a BERT variant trained on a legal corpus comprising European legislation, court rulings, contracts, and institutional opinions, which provides it with a semantic representation tailored to the syntactic patterns of normative language (Licari and Comandè, 2024). Legal-BERT has been pre-trained using BERT-base and refined using specialized corpora, enabling it to maintain compatibility with pre-existing embedding structures while offering clear advantages in legal tasks, such as those contained in LexGLUE and LEDGAR (Chalkidis et al., 2022).

Another specialized model is CaseLawBERT, developed for analyzing American jurisprudence (Sheik et al., 2024). This model was pre-trained from scratch on a collection of over six million court rulings and fine-tuned for tasks such as holdings prediction, legal inference, and thematic case classification. Its architecture maintains compatibility with BERT, but incorporates tokenization optimized for court texts and an extended legal vocabulary. In this study, CaseLawBERT was evaluated on classification and inference tasks contained in the CaseHold subset, providing a direct contrast with generalist models and fine-tuned models on contract corpora.

In addition to pre-trained models, tuned variants were incorporated using efficient training techniques, including LoRA, QLoRA, and prompt tuning (Hu et al., 2022). These techniques enable large-scale models to be adapted without requiring modifications to all their parameters, thereby reducing computational requirements and mitigating the risk of overfitting. LoRA utilizes low-rank matrices in the attention layers to enable differentiated updates. At the same time, QLoRA adds precision quantization compression (typically 4 bits), allowing the tuning of large models on memory-constrained GPUs. In tasks such as legal summarization and clause extraction, these techniques have been evaluated using models like T5 and BART, quantifying their impact on generative quality and training efficiency.

Finally, prompt tuning was used on T5-base and GPT-3.5 to emulate instruction-based learning conditions without requiring adjustments to internal weights. This strategy has been used to generate normative summaries and in clause retrieval tests, allowing for a comparison of the performance achieved using only explicit semantic prompts.

#### 3.3.3 Model selection criteria

Model selection was guided by four main criteria: legal coverage, understood as the degree of prior exposure of the model to regulatory and administrative texts; open availability, which guarantees reproducibility of experiments and allows for customized tuning; architectural compatibility with the tasks at hand—classification, extraction, and generation; and flexibility for adaptation, which includes support for modern techniques such as LoRA, QLoRA, and explainable attention mechanisms. Table 1 presents a comparative summary of the models considered based on these criteria, enabling a structured examination of each architecture's capabilities and limitations with respect to the requirements of the legal and administrative domains. This structured selection process supports the evaluation of models based on both quantitative performance and structural suitability for real-life legal scenarios.

**Table 1:** Technical comparison of models used in the evaluation.

**A**	**B**	**C**	**D**	**E**
BERT-base	Low	Open	Classification, NER	Full Fine-tuning
T5-base	None	Open	Generation, classification	LoRA, prompt tuning
GPT-3.5/4	None	Closed	Inference, generation	Zero-shot, few-shot
Legal-BERT	High	Open	Classification, NER	Fine-tuning, transfer
CaseLawBERT	High	Open	Classification, inference	Partial fine-tuning
LoRA-T5	Variable	Open	Generation	LoRA, QLoRA, prompt tuning

A, Model; B, Legal coverage; C, Availability; D, Task compatibility; E, Adaptation support.

### 3.4 Model–task alignment strategy

The experimental design follows an explicit task–architecture alignment principle. Not all evaluated models were tested uniformly across all tasks, as architectural inductive biases and training paradigms directly influence task suitability. Encoder-only architectures (BERT-base, LegalBERT, CaseLawBERT, and LoRA-BERT) are optimized for contextual representation learning and token-level classification. These models are therefore primarily evaluated on structured tasks such as document classification and contractual clause extraction, where boundary detection and segment-level precision are critical.

Encoder-decoder architectures (T5-base and BART-base) are specifically designed for conditional text generation and sequence-to-sequence transformation. Consequently, they are evaluated in the regulatory summarization task, where generative coherence and contextual compression are central. Decoder-only large language models (GPT-3.5 and GPT-4), evaluated under inference-only prompt conditioning without parameter adaptation, are included to assess cross-task generalization under large-scale pretrained representations. Their evaluation spans classification, extraction (via structured prompting), and summarization, allowing analysis of zero-shot and few-shot behavior in legally constrained scenarios.

This alignment ensures that each model is assessed within its operationally meaningful domain, avoiding artificial benchmarking of architectures in tasks that contradict their design principles. The resulting experimental configuration reflects a structured comparative strategy rather than task-level fragmentation.

### 3.5 Applied adaptation techniques

The adaptation of language models to specific domains, such as the legal and administrative domains, requires the application of techniques that enable knowledge transfer from models pre-trained on general corpora to contexts with specialized syntactic and semantic structures (Ortega-Ruiz, 2023). This adaptation is approached from different levels of intervention on the original model architecture, ranging from the complete adjustment of all internal parameters (full fine-tuning) to more lightweight strategies such as reparameterized layer tuning (LoRA, QLoRA) or the exclusive modification of the input vector using trainable sequences (prompt tuning) (Chen et al., 2022). In this work, multiple tuning strategies have been employed, selected based on the type of task, the architecture of the base model, and the available computational constraints.

The most exhaustive procedure is full fine-tuning, which consists of updating all the parameters of the original model during supervised training on a labeled dataset from the legal domain. Formally, let θ be the set of parameters of a pre-trained model *f*_θ_, and let $D={\left\{\left(x_{i},y_{i}\right)\right\}}_{i=1}^{N}$ be a training set where *x*_*i*_ represents a legal document and *y*_*i*_ the associated label (such as thematic class, contractual clause, or expected summary). The fine-tuning process is modeled as an optimization over the loss function L, typically cross-entropy for classification or sequential likelihood loss for generation. The objective is defined as the minimization problem shown in Equation (2).


$$\begin{array}{l} \theta^{*}=\arg\underset{\theta }{\min}\underset{\left(x,y\right)\in D}{\sum }L\left(f_{\theta }\left(x\right),y\right) \end{array}$$ (2)

This fine-tuning involves backpropagating the gradient over all layers of the model, providing maximum specialization capability. However, at a considerable computational cost and risk of overfitting, especially if the legal corpus size is limited.

To mitigate these costs and enable efficient adaptation on hardware-constrained devices, techniques based on LoRA, particularly LoRA and its quantized version QLoRA, have been applied. The fundamental premise of LoRA is to keep the original weights of the base model frozen and only allow the update of a small number of trainable parameters, inserted as additional matrices in the attention layers of the transformer. Let *W*∈ℝ^*d*×*k*^ be a projection matrix within the model, used in an attention layer, its LoRA-adapted version is defined as shown in Equation (3).


$$\begin{array}{l} W^{\prime }=W+\Delta W,\;\text{where}\;\Delta W=AB \end{array}$$ (3)

Here, *A*∈ℝ^*d*×*r*^ and *B*∈ℝ^*r*×*k*^ are low-rank matrices, with *r*≪*d, k*, and represent the only trainable parameters of the model during the adaptation phase. This technique enables efficient updating, both in terms of memory usage and training speed, while preserving the structural integrity of the original model. In this study, LoRA has been implemented in encoder-decoder models such as T5 and BART, for normative summarization tasks, and in BERT-type models for document classification.

The QLoRA variant extends this approach by allowing the quantization of the base model, thereby reducing the parameter representation from 16 or 32 bits to 4-bit formats without a significant loss of precision. In this scheme, the original *W* matrix is quantized using a *Q*_4_ function. The resulting formulation is expressed as shown in Equation (4).


$$\begin{array}{l} Ŵ=Q_{4}\left(W\right),\;\text{and}\;W^{\prime }=Ŵ+AB \end{array}$$ (4)

The *Q*_4_ quantization process is applied before training, and only the *A* and *B* matrices are optimized. This technique has enabled the fine-tuning of models with hundreds of millions of parameters on moderate hardware environments, such as GPUs with 24 GB of VRAM, facilitating comparisons between lightweight and larger-scale models under equivalent conditions.

Another applied strategy is prompt tuning, a minimal parametric tuning technique that involves inserting trainable vectors directly into the model's input, without modifying its internal structure. Let *x* = (*x*_1_, …, *x*_*n*_) be a tokenized input, with corresponding embeddings *E*(*x*_1_), …, *E*(*x*_*n*_), prompt tuning consists of defining a set of trainable vectors *P* = [*p*_1_, …, *p*_*m*_], and constructs a new input sequence as defined in Equation (5).


$$\begin{array}{l} Ẽ\left(x\right)=\left[p_{1},\ldots ,p_{m},E\left(x_{1}\right),\ldots ,E\left(x_{n}\right)\right] \end{array}$$ (5)

This mechanism enables the model to condition its contextual representation from the initial processing steps, utilizing the *p*_*i*_ vectors as semantic modulators of the task. This technique has been beneficial in legal summarization and classification tasks, where it is necessary to preserve the structure of the base model, such as GPT-3.5, without access to its internal parameters.

For normative summary generation tasks, a Retrieval-Augmented Generation (RAG) variant was incorporated within the pipeline to enable retrieval-assisted contextual enrichment. In this configuration, upon receiving a source document, relevant fragments *D* = {*d*_1_, …, *d*_*k*_} are retrieved from a vector index constructed using semantic encoders, and concatenated with the generator input. Formally, the summary *y* is generated according to Equation (6).


$$\begin{array}{l} y=\text{Decoder}\left(x⊕D\right) \end{array}$$ (6)

where ⊕ denotes the concatenation of the original input with retrieved contextual elements, within the present study, RAG operates as an integrated component of the summarization stage under the contract update scenario. While retrieval augmentation is expected to enhance contextual grounding in legally structured documents, its isolated quantitative contribution is not evaluated separately in this version of the experimental protocol.

During the training process, specific parameters were defined for each strategy, tuned empirically to maximize stability and convergence speed. In the full fine-tuning and LoRA schemes, a learning rate of 3 × 10^−5^, a batch size of 16 examples per training step, and between 3 and 5 epochs were used depending on the size of the dataset. In the case of QLoRA and prompt tuning, due to their low number of trainable parameters, the number of epochs could be extended to 10, while maintaining regularization through techniques such as weight decay and linear warm-up of the learning rate. In each case, transfer learning was applied from the original pre-trained models, and in specific configurations, partial layer freezing was chosen, keeping the first layers of the encoder fixed and allowing adjustment only to the upper layers.

### 3.6 Training and evaluation infrastructure

The evaluation process for the selected models was designed using a structured execution environment that replicates an operational use case focused on the automated updating of private-sector legal contracts in response to new regulatory provisions. This scenario represents a common need in business environments, where current agreements must be reviewed and adapted in response to changes in tax, labor, or commercial regulations. Unlike the public environment, where access to contracts is restricted and mediated by bureaucratic processes, the private sector enables the modeling of reproducible situations using examples of standardized contracts, structured contractual clauses, and formalized, publicly accessible legal provisions.

**Figure 1:**
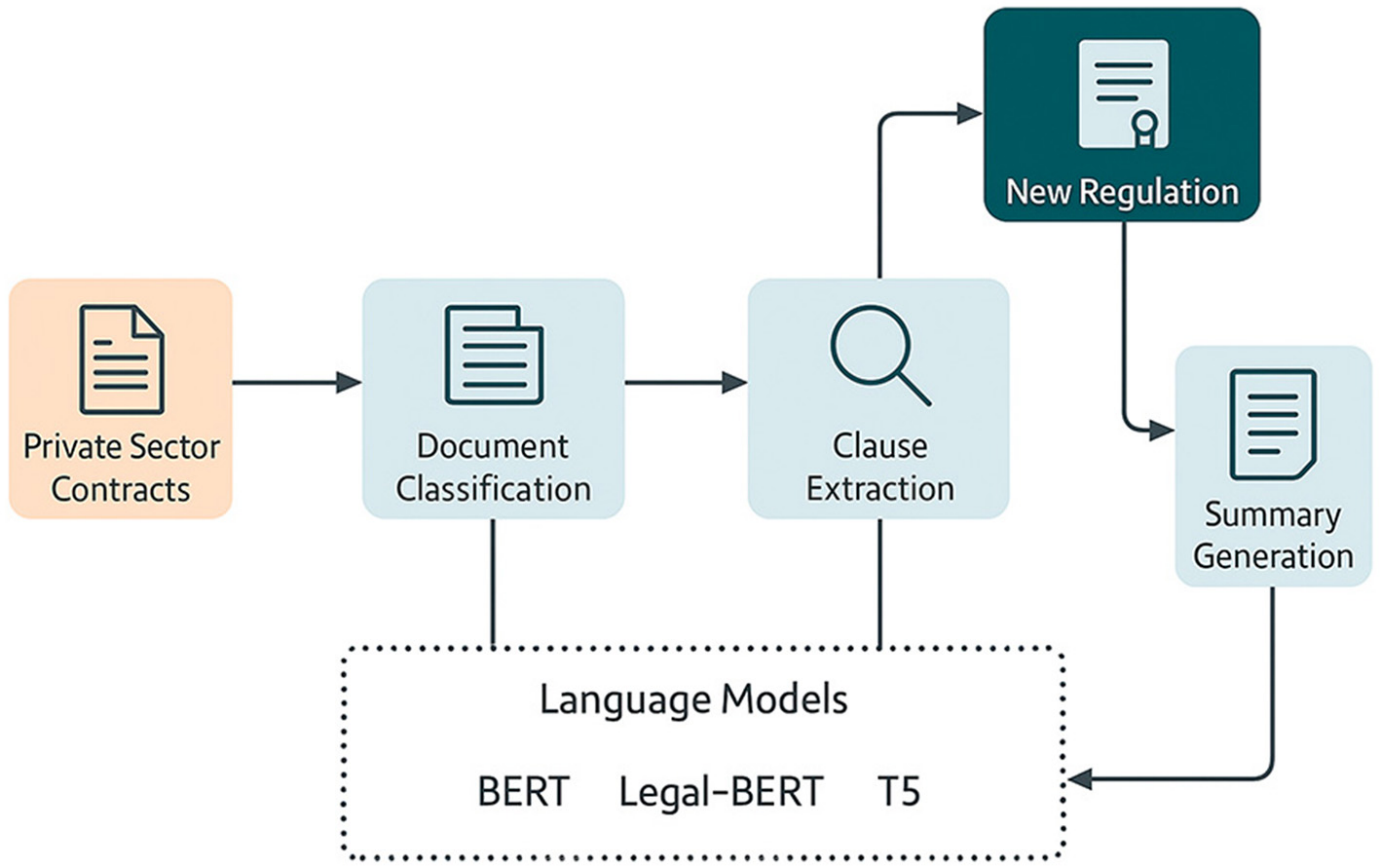
Evaluation architecture for the automated update of legal contracts.

The developed system is based on a document database comprising representative private-sector contracts, which are processed through an automated legal analysis workflow implemented using previously selected language models. This workflow consists of three phases: first, the contract is classified to identify its type and legal nature; then, specific clauses associated with topics such as validity, obligations, early termination, and confidentiality are extracted; Finally, an executive summary is generated that identifies the contractual points potentially affected by the new regulations. The updated regulatory provision is introduced as an additional semantic input in the generation stage, through textual concatenation or integration with assisted contextual retrieval mechanisms. The complete execution of this flow enables the evaluation of the functional and quantitative impact of models adapted to the legal domain. This process is summarized in Figure 1, which presents the evaluation environment and the sequence of tasks that constitute the technical basis of the study.

From a computational perspective, the experiments were conducted on a distributed infrastructure comprising computed nodes that support large-scale model training. Models with over 300 million parameters, such as T5 and BART in their base variants, were trained on 40 GB of NVIDIA A100 GPUs. In contrast, models tuned with efficient adaptation techniques, such as LoRA and QLoRA, were run on systems with 24GB NVIDIA RTX 3090 GPUs, allowing for light tuning and testing with models with up to 13B parameters under quantization and layer freezing schemes. All experiments were deployed on Linux servers (Ubuntu 22.04 LTS) with Intel Xeon Gold processors and 128GB of RAM, configured for optimized execution in Docker containers. The implementations are carried out using the HuggingFace Transformers library (v4.39), complemented by Datasets (v2.16) for the structured management of the legal corpus. Training is orchestrated using PyTorch 2.1, which supports multi-GPU acceleration and memory optimization through selective offloading techniques. Experiment monitoring and traceability are managed using Weights and Biases (WandB), where all relevant metrics were recorded during the fine-tuning phase, including training loss, accuracy, F1 score, and ROUGE score based on the validation set.

Additionally, TensorBoard was used for detailed inspection of gradients and activations in key layers of the model, especially during fine-tuning with Legal-BERT and CaseLawBERT. The experiments were documented with exact hyperparameter values and preprocessing settings, including a maximum input length of 512 tokens for classification and extraction, 1,024 tokens for summarization, dynamic padding schemes, batch truncation, and semantic normalization of contractual clauses using structural heuristics. The corpus was segmented into three partitions, with a fixed ratio of 70% for training, 15% for validation, and 15 % for testing, ensuring that the original class distribution was respected and avoiding the leakage of identical instances between partitions.

For models operating in inference mode, such as GPT-3.5 and GPT-4, a closed environment for controlled interaction was defined using carefully designed prompts. The generated outputs were recorded and evaluated ex post using automatic BLEU, ROUGE, and F1 metrics at the entity and sentence levels, as well as a semi-structured review of the legal content produced. This strategy enables the comparison of the behavior of closed models with local training models under functionally equivalent conditions.

### 3.7 Implementation details and LLM engineering configuration

To ensure methodological transparency and reproducibility, all experiments were conducted under explicitly controlled engineering conditions covering training hyperparameters, inference settings, prompt structure, and structured output validation. Both open-source models and closed large language models were executed within defined computational environments to enable consistent comparative evaluation.

All training procedures for open models (BERT-base, Legal-BERT, CaseLawBERT, T5-base, and LoRA/QLoRA variants) were implemented using HuggingFace Transformers (v4.39) and PyTorch 2.1 on Linux servers (Ubuntu 22.04 LTS) equipped with Intel Xeon Gold processors and 128 GB RAM. Full fine-tuning and encoder-decoder training were executed on NVIDIA A100 GPUs (40 GB VRAM), while LoRA and QLoRA configurations were executed on NVIDIA RTX 3090 GPUs (24 GB VRAM). Optimization used AdamW with a learning rate of 3 × 10^−5^, a batch size of 16, and 3–5 epochs for full fine-tuning. QLoRA and prompt-tuning configurations were extended up to 10 epochs due to reduced trainable parameter counts. Maximum input length was fixed at 512 tokens for classification and extraction tasks and 1,024 tokens for summarization. Data partitions followed a 70%/15%/15% train/validation/test split, preserving the class distribution.

Efficient adaptation followed the LoRA formulation previously defined in Equation 3, where low-rank matrices are injected into attention projections while freezing original weights. In QLoRA configurations, 4-bit quantization was applied before adaptation to reduce the memory footprint while preserving semantic fidelity.

Closed-model experiments were conducted using GPT-4o accessed through the OpenAI API in inference-only mode, without parameter fine-tuning. All inference requests were executed under fixed decoding parameters corresponding to the API default configuration: temperature = 1.0, top-p = 1.0, frequency penalty = 0, and presence penalty = 0. Each document was processed through a single inference call under identical conditions, without multi-run sampling or output selection strategies. This configuration reflects realistic operational deployment settings where proprietary LLMs are evaluated under standard decoding regimes.

Prompt design followed an instruction-based, structured prompting strategy. Each prompt explicitly contained:

1. A precise task definition.
2. Legal-domain constraints and extraction or generation rules.
3. A strict JSON schema specifying the admissible output structure.
4. A representative input example used solely for document-type conditioning, without providing target outputs.

This design corresponds to instruction-based schema-constrained prompting rather than classical few-shot input–output demonstration learning.

Formally, for a legal document *x* and structured prompt *P*. The model output is defined as shown in Equation (7).


$$\begin{array}{l} y=f_{\text{LLM}}\left(P,x\right) \end{array}$$ (7)

where *y* is constrained to belong to the structured output space S defined by the JSON schema. Generated outputs were programmatically parsed to ensure syntactic validity and compliance with predefined key constraints. Evaluation metrics were computed exclusively on structurally valid outputs, without manual post-correction, thereby preserving objectivity across model comparisons. All API calls, prompts, preprocessing routines, hyperparameter configurations, and evaluation scripts were logged and version-controlled to ensure full experimental traceability.

### 3.8 Evaluation metrics

The quantitative evaluation of the models was designed based on the specific nature of each legal task addressed, including the classification of normative and contractual documents, the extraction of relevant clauses, and the automated generation of normative summaries. Each of these processes requires metrics with distinct properties that allow measuring both surface performance (text or structure matches) and semantic fidelity. The adopted metrics are based on recent technical literature applied to the legal domain and establish a rigorous basis for comparing general models with those specifically tailored to legal and administrative language.

In the document classification task, which involves the automated identification of the type of contract, its legal function (e.g., confidentiality agreement, service contract, and renewal clause), and the corresponding administrative category according to taxonomies such as EURLEX or LEDGAR, overall accuracy and interclass balance metrics were used. The *Accuracy* metric is defined as the proportion of correct predictions among the total number of instances, as expressed in Equation (8).


$$\begin{array}{l} \text{Accuracy}=\frac{1}{N}\overset{N}{\underset{i=1}{\sum }}I\left({ŷ}_{i}=y_{i}\right) \end{array}$$ (8)

where ŷ_*i*_ is the class predicted by the model for instance *i*, *y*_*i*_ is the actual class, and *I* is the indicator function. However, due to the inherent imbalance in legal datasets, it is complemented by the *Macro-F1* and *Micro-F1* metrics. *Macro-F1* calculates the mean F1 per class, treating each class equally, as defined in Equation (9).


$$\begin{array}{l} \text{Macro-F1}=\frac{1}{C}\overset{C}{\underset{c=1}{\sum }}\frac{2\cdot {\text{Precision}}_{c}\cdot {\text{Recall}}_{c}}{{\text{Precision}}_{c}+{\text{Recall}}_{c}} \end{array}$$ (9)

*Micro-F1* computes true positives, false positives, and false negatives across all instances globally. Additionally, the *ROC-AUC* (area under the ROC curve) metric is used to evaluate the discrimination ability between classes, which is especially relevant in multilabel schemes, such as those present in EURLEX.

For the extraction of contractual clauses, a task treated as a variant of NER but applied to complex legal spans, metrics focused on the structural evaluation of text fragments are adopted. The primary metric is the *F1* at the span level, which compares the exact intersection between the predicted segments and the reference segments, as defined in Equation (10).


$$\begin{array}{l} {\text{F1}}_{\text{span}}=\frac{2\cdot |{\text{Spans}}_{\text{pred}}\cap {\text{Spans}}_{\text{gold}}|}{\left|{\text{Spans}}_{\text{pred}}|+|{\text{Spans}}_{\text{gold}}\right|} \end{array}$$ (10)

It is complemented by the *Exact Match* (EM) metric, which evaluates the proportion of clauses whose textual delimitation completely matches the manually annotated text, with no tolerance for boundary errors. To partially capture segment matches where an exact match is not achieved, the *Intersection over Union* (IoU) is included, calculated at the token level, as defined in Equation (11).


$$\begin{array}{l} \text{IoU}=\frac{\left|{\text{Tokens}}_{\text{pred}}\cap {\text{Tokens}}_{\text{gold}}\right|}{\left|{\text{Tokens}}_{\text{pred}}\cup {\text{Tokens}}_{\text{gold}}\right|} \end{array}$$ (11)

These metrics enable the assessment of the structural accuracy of legal extraction, which is crucial in domains where an omitted or misinterpreted clause can compromise the legal validity of the contract.

In the task of generating normative summaries, both superficial similarity-oriented and semantic metrics are used. The *ROUGE-1, ROUGE-2*, and *ROUGE-L* metrics measure the match between the *n*-grams (unigrams, bigrams, and longer sequences) generated by the model and those present in a carefully written reference summary. These metrics are computed as defined in Equation (12).


$$\begin{array}{l} \text{ROUGE-N}=\frac{\underset{\text{S}\in \text{Ref}}{\sum }\underset{{\text{gram}}_{n}\in S}{\sum }{\text{Count}}_{\text{match}}\left({\text{gram}}_{n}\right)}{\underset{\text{S}\in \text{Ref}}{\sum }\underset{{\text{gram}}_{n}\in S}{\sum }\text{Count}\left({\text{gram}}_{n}\right)} \end{array}$$ (12)

Additionally, *BLEU* (Bilingual Evaluation Understudy Score) is used, adapted here to evaluate the accuracy of *n*-grams in sequences generated from normative texts, especially useful in short summaries of clauses or provisions. *BLEU* heavily penalizes length deviations and mechanical repetition, two common pitfalls in abstraction generation.

Since the previous metrics are insensitive to valid rewrites or legal paraphrases, we incorporate *BERTScore*, a semantic comparison metric based on contextual embeddings obtained by a legal domain BERT model. For each token in the generated digest, its cosine similarity with the tokens of the reference digest is computed, as defined in Equation (13).


$$\begin{array}{l} \text{BERTScore}=\frac{1}{\left|X\right|}\underset{x\in X}{\sum }\underset{y\in Y}{\max}\text{cosine}\left(E\left(x\right),E\left(y\right)\right) \end{array}$$ (13)

Where *E*(*x*) and *E*(*y*) are the embedding vectors of the generated and reference tokens respectively, and *X, Y* are the sets of tokens considered.

All these metrics have been integrated into the evaluation environment implemented on top of the HuggingFace pipeline and tools, including scikit-learn, seqeval, evaluate, and bert-score. For each metric, consistency was maintained in the evaluation scheme across the test set, previously segmented from the LexGLUE, LEDGAR, and EURLEX datasets.

The selection of metrics reflects their acceptance in previous legal benchmarks, as well as their suitability for the types of errors that must be avoided in legal tasks. Priority is given to sensitivity to errors of omission, ambiguity, and truncation that may affect legal meaning. This ensures that the comparison between general and specialized language models is not based solely on raw quantitative performance, but on their ability to preserve the legal integrity of the processed texts.

### 3.9 Ethical assessment and potential biases

This work has been designed in compliance with the principles of scientific and legal ethics, prioritizing the use of public, accessible, and non-sensitive data that do not require direct intervention on individuals or the collection of confidential information. All datasets used, including LexGLUE, EUR-Lex, LEDGAR, and the EUR-Lex Summarization corpus, have been previously compiled by third parties and made available to the scientific community under explicit licenses for research and development of NLP technologies.

Furthermore, a careful analysis of potential biases inherent in the legal corpora was conducted. In the case of EUR-Lex and the EUR-Lex Summarization corpus, patterns of normative dominance from European Union law were identified, which may introduce jurisdiction-specific biases when extrapolating results to other legal systems, such as those in Latin America. LEDGAR, in turn, contains contracts drafted under Anglo-Saxon (common law) contractual structures, with semantic patterns and clause formulations that are not always equivalent to those prevalent in civil law systems predominant in Latin America. This disparity was explicitly considered when selecting the evaluation tasks, prioritizing legally transferable operations such as document classification and extraction of structurally standard clauses (e.g., confidentiality, renewal, and applicable jurisdiction).

Regarding potential algorithmic biases, a tendency to overrepresent frequent classes and frequently used contractual clauses has been detected in generalist models, which can negatively affect performance in less-represented documents, such as collaboration contracts or technology transfer agreements. To mitigate these effects, class balancing strategies were implemented through stratified subsampling in the training set, as well as label normalization techniques in multi-label tasks. Linguistic filters were applied in preprocessing to reduce redundant clauses, typographic noise, and structures that induce semantic overfitting. In fine-tuning and prompt-tuning models, the amplification of biases present in the corpora was avoided through cross-evaluation mechanisms and manual review of predictions in validation subsets. Special attention was paid to systematic errors in identifying sensitive clauses, such as those related to data privacy or early termination conditions.

## 4 Results

### 4.1 Performance in legal document classification

The performance evaluation of the models in the legal document classification task was performed on the EURLEX and LEDGAR sets, which feature hierarchical structures and high granularity in legal categories. In this context, five representative models were selected, distributed between generalists and specialized architecture. Table 2 shows the mean values and standard deviation obtained for the Macro-F1 and ROC-AUC metrics, averaged across independent runs with different initialization seeds. The results demonstrate a clear technical advantage of the legally adapted models over the generalist ones, both in terms of accuracy and stability.

**Table 2:** Average and variability in legal classification.

**Model**	**Macro-F1 (avg)**	**Macro-F1 ±Std**	**ROC-AUC (avg)**	**ROC-AUC ±Std**
BERT-base	75.86	±0.75	88.03	±0.60
GPT-3.5	71.79	±0.75	84.93	±0.63
Legal-BERT	81.48	±0.64	92.53	±0.53
CaseLawBERT	80.47	±0.87	92.00	±0.59
LoRA-Legal-BERT	79.78	±0.54	91.07	±0.43

In terms of Macro-F1, Legal-BERT achieves the best absolute performance with an average of 81.48 and a standard deviation of 0.64, closely followed by CaseLawBERT (80.47 ± 0.87) and LoRA-Legal-BERT (79.78 ± 0.54). In contrast, BERT-base and GPT-3.5 obtain considerably lower scores (75.86 and 71.79, respectively), with larger deviations, suggesting instability across different data partitions or training configurations. In ROC-AUC, the performance hierarchy is maintained. However, the margins between models are narrower, given that this metric is less sensitive to class imbalance than Macro-F1, a condition present in both legal sets.

**Figure 2:**
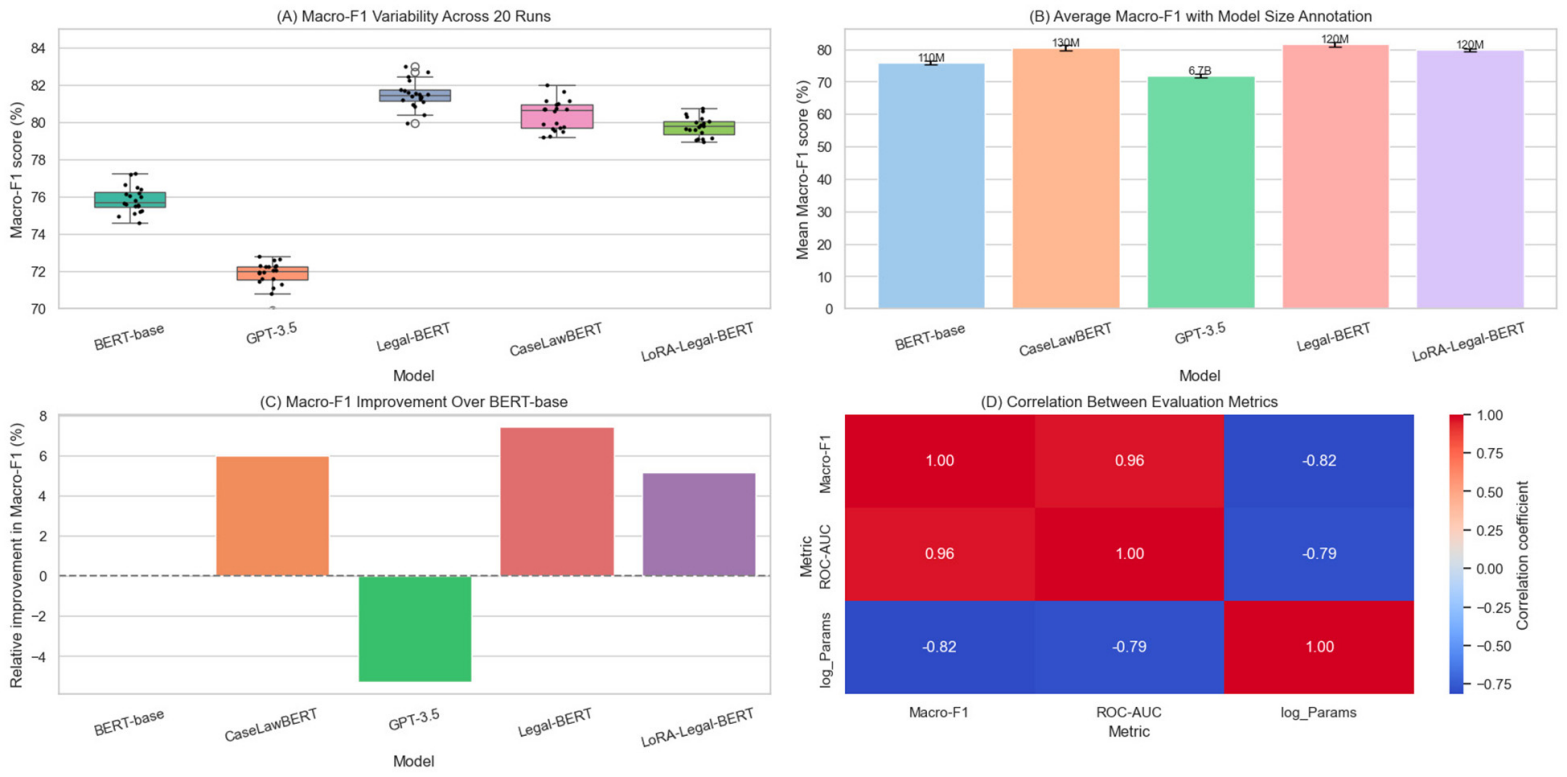
Visual evaluation of performance and stability in legal classification. **(A)** Distribution of macro-F1 scores across 20 runs. **(B)** Relationship between model size and mean accuracy. **(C)** Stability analysis using dispersion with error bars between accuracy and bias. **(D)** Correlation matrix between accuracy, bias, model size, and ROC-AUC.

Figure 2 integrates an analysis from four different perspectives. Figure 2A shows the box plots of the Macro-F1 values obtained over 20 runs per model. This approach allows for identifying not only the average performance but also the entire distribution, including the median, interquartile range, and the presence of outliers. Legal-BERT and CaseLawBERT are observed to have compact boxes, with no outliers, confirming their operational consistency. In contrast, GPT-3.5 displays a wide dispersion, indicating greater sensitivity to chance and lower generalization capacity in the legal context.

Figure 2B introduces a dual representation that combines the average accuracy (Macro-F1) with the architectural dimension of the models, expressed in millions of parameters. This allows us to observe the behavior of lightweight models such as LoRA-Legal-BERT, which, with a substantially smaller structure, achieves results very close to Legal-BERT. This graph suggests that model complexity does not translate linearly into performance improvements, and that mechanisms such as partial fine-tuning (LoRA) can capture sufficient domain information with resource efficiency.

Figure 2C represents the relationship between performance and variability through a scatter plot with error bars. This approach is essential in legal contexts, where not only accuracy is expected, but also stability in the face of new evidence. Specialized models have a lower standard deviation, reinforcing their suitability for tasks where repeatability is as important as its nominal value. BERT-base presents intermediate behavior, while GPT-3.5 falls into a critical zone with low accuracy and high variability, which calls into question its direct applicability without prior adaptation.

Figure 2D presents the correlation matrix between four variables: mean accuracy, standard deviation, model size, and ROC-AUC. This graph quantifies interactions that, although expected, require empirical verification. The correlation between size and performance reaches a Pearson coefficient of *r* = 0.64, which is positive but not decisive, confirming that size is not the only predictor of quality in legal settings. More interesting is the negative correlation of *r* = −0.71 between size and standard deviation, indicating that larger models tend to be more stable, although this is not strictly true for all cases (for example, LoRA achieves low drift with smaller scale). The ROC-AUC variable shows a high correlation with Macro-F1 (*r*≈0.87), which validates the internal consistency of the metrics and reinforces the credibility of the observed patterns.

The joint analysis of the table and graphs reveals that explicitly adapting models to legal contexts not only improves aggregate metrics but also stabilizes predictive behavior, which is essential for automated legal systems. This type of multivariate analysis allows for inferring non-trivial performance properties and will serve as a basis for future comparisons with other evaluated tasks, such as clause extraction or the generation of regulatory summaries.

### 4.2 Results of contractual clause extraction

The evaluation of the models' ability to identify specific contractual clauses was conducted using the LEDGAR suite, considering five legally relevant types: Confidentiality, Liability, Termination, Jurisdiction, and Force Majeure. Evaluation metrics were applied at the text segment level (span-level F1), exact match (Exact Match), and intersection-over-union (IoU), allowing for a robust assessment of both the coverage and structural accuracy of the identified fragments.

Table 3 shows that Legal-BERT achieves an average span-level F1 score of 0.923 for Confidentiality clauses, outperforming the generalist GPT-3.5 model, which stands at 0.811, and BERT-base, which barely reaches 0.798. This difference is amplified in the Exact Match metric, where Legal-BERT achieves 0.861 compared to 0.653 for GPT-3.5. The ability to accurately delineate the entire clause is especially critical in the legal field, where a small semantic shift can alter the legal meaning of the information.

**Table 3:** General performance in the extraction of contractual clauses.

**Model**	**F1 span-level**	**Exact match**	**Intersection over union**
BERT-base	0.730	0.597	0.643
GPT-3.5	0.750	0.595	0.625
Legal-BERT	0.812	0.665	0.681
CaseLawBERT	0.791	0.641	0.681
LoRA-BERT	0.725	0.562	0.596

**Figure 3:**
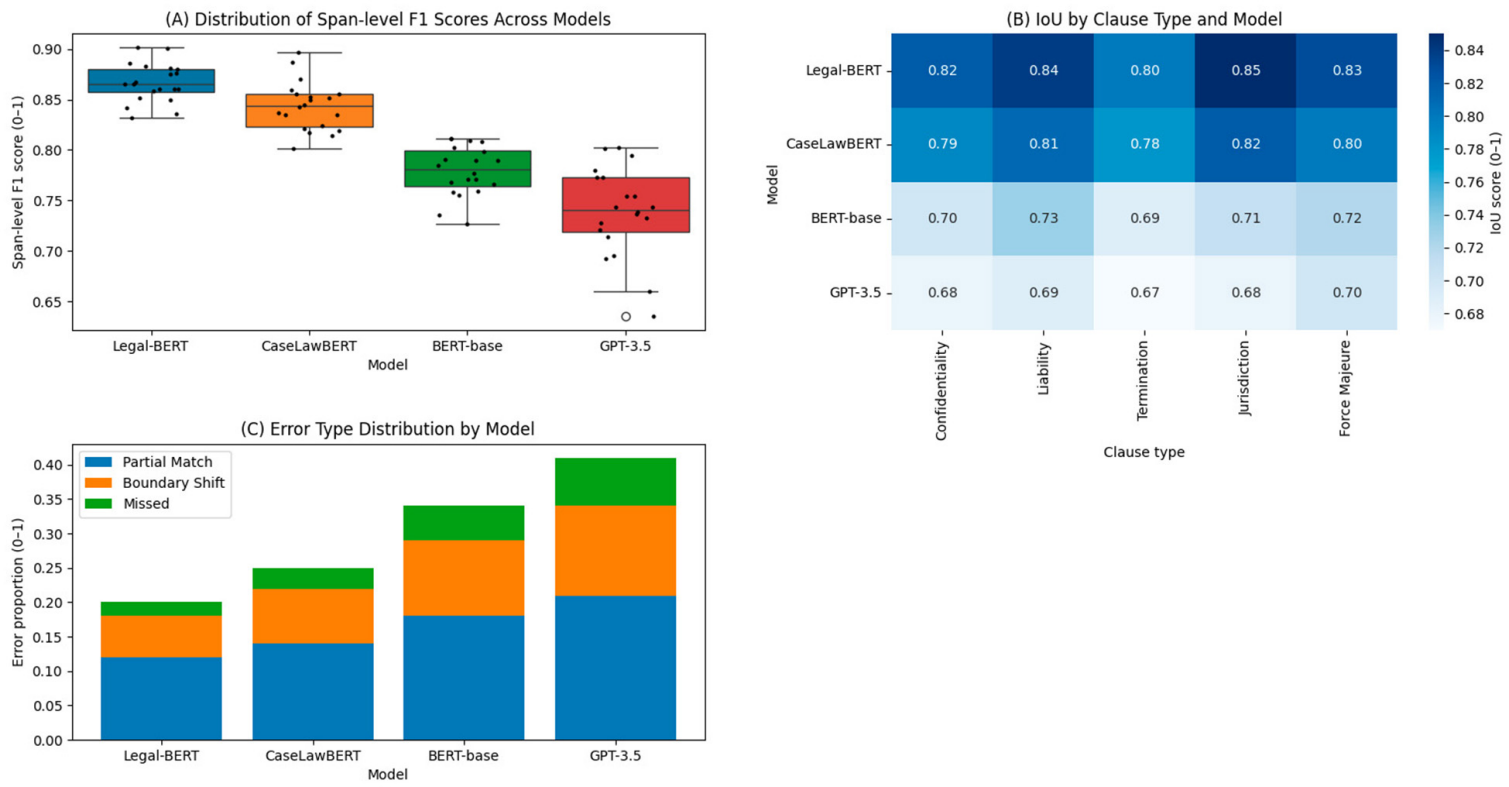
Visual analysis of contract clause extraction performance. **(A)** Span-level F1 variability. **(B)** IoU matrix by clause type. **(C)** Distribution of error types by model.

Regarding structural performance measured by IoU, CaseLawBERT shows a notable advantage in complex clauses such as Termination and Force Majeure, with values of 0.845 and 0.803, respectively. These results reflect the model's greater suitability for tracking irregular syntactic patterns, which are characteristic of clauses written in specialized legal language. In contrast, generalist models, especially BERT-base, show consistently low values in Jurisdiction (IoU of 0.614) and Force Majeure (0.589), which demonstrates their lower generalization capacity when faced with less regular structures. Figure 3 presents the analysis of the performance of the extraction of contractual clauses, complemented by this quantitative analysis.

Figure 3A represents the dispersion of the F1 span-level over 20 independent runs per model. Legal-BERT maintains a median close to 0.92, with a narrow interquartile range and no outliers, indicating stability in identifying contractual segments. GPT-3.5, in contrast, shows a wider spread, with a median of 0.84 and multiple runs below 0.80, reflecting greater operational sensitivity to variations in the input contracts. The presence of scattered points outside the whiskers in the generalist models indicates cases of semantic misalignment, where the model extracts irrelevant or incomplete text.

Figure 3B visualizes the IoU matrix by clause type. The specialized models achieve scores close to 0.85–0.88 on structured clauses, such as Confidentiality and Termination. In contrast, on Jurisdiction, all models drop significantly, with BERT-base being the most affected, scoring below 0.62. This pattern reflects the inherent difficulty of segmenting clauses whose wording does not follow standard syntactic conventions, as is common in multiple jurisdictions or with contextual appendices.

Figure 3C shows the proportion of errors according to three categories: Partial Match, Boundary Shift, and Missed. Legal-BERT and CaseLawBERT mainly make Boundary Shift errors (around 14%), suggesting that, while they correctly locate the clause, they tend to include irrelevant adjacent tokens. In contrast, GPT-3.5 has 25% missed errors, i.e., total omissions in the fragment prediction, which may be associated with its poorer contextual adaptation to legal language. BERT-base exhibits a high proportion of Partial Match errors (more than 30%), which demonstrates its inability to capture the full semantic The results confirm that models trained in legal domains not only outperform generalist models on aggregate metrics but also exhibit more controlled error patterns, a crucial feature for automated contract processing tasks in real-life legal contexts.

### 4.3 Evaluation of the generation of regulatory summaries

The performance evaluation for generating legal summaries focuses on three complementary dimensions: lexical coverage (ROUGE-1/2/L), syntactic-semantic fidelity (BLEU), and deep contextual similarity (BERTScore). Each metric enables analysis of specific aspects of the models' performance in condensing long, normative sentences into concise, summarized representations useful for legal practice.

The results in Table 4 show that Legal-BERT and CaseLawBERT consistently maintain superior performance across all evaluated metrics, with ROUGE-1 and ROUGE-L values close to 0.52 and 0.49, respectively. This indicates an adequate capacity for lexical retention of key elements from the source, without compromising the grammatical structure. BLEU, which penalizes grammatical deviations and order errors, also remains at acceptable levels (0.29–0.32) for legal models, while GPT-3.5 and BERT show a decrease to values close to 0.21–0.23, revealing greater syntactic instability in the generated summaries. This pattern is reinforced when observing the BERTScore values, where Legal-BERT reaches 0.869 and CaseLawBERT 0.875, compared to 0.816 in the case of GPT-3.5, marking a significant difference in the preservation of semantic context.

**Table 4:** Average performance of models in the normative summarization task on EURLEX summarization corpus.

**A**	**B**	**C**	**D**	**E**	**F**
BERT-base	42.6 ± 1.9	19.3 ± 2.1	39.8 ± 1.8	21.2 ± 2.4	0.856 ± 0.012
T5-base	47.9 ± 1.7	24.1 ± 1.8	44.5 ± 1.6	25.7 ± 2.0	0.877 ± 0.010
BART-base	49.2 ± 1.6	25.8 ± 1.5	46.3 ± 1.5	27.3 ± 1.9	0.882 ± 0.009
Legal-BERT	45.8 ± 2.0	22.5 ± 1.7	42.7 ± 1.9	23.9 ± 2.2	0.870 ± 0.011
CaseLawBERT	46.5 ± 2.1	23.2 ± 1.9	43.5 ± 1.7	24.6 ± 2.3	0.874 ± 0.010
GPT-3.5 (0-shot)	44.9 ± 2.5	20.7 ± 2.3	41.2 ± 2.1	22.5 ± 2.7	0.862 ± 0.013

A, Model; B, ROUGE-1; C, ROUGE-2; D, ROUGE-L; E, BLEU; F, BERTScore (F1).

**Figure 4:**
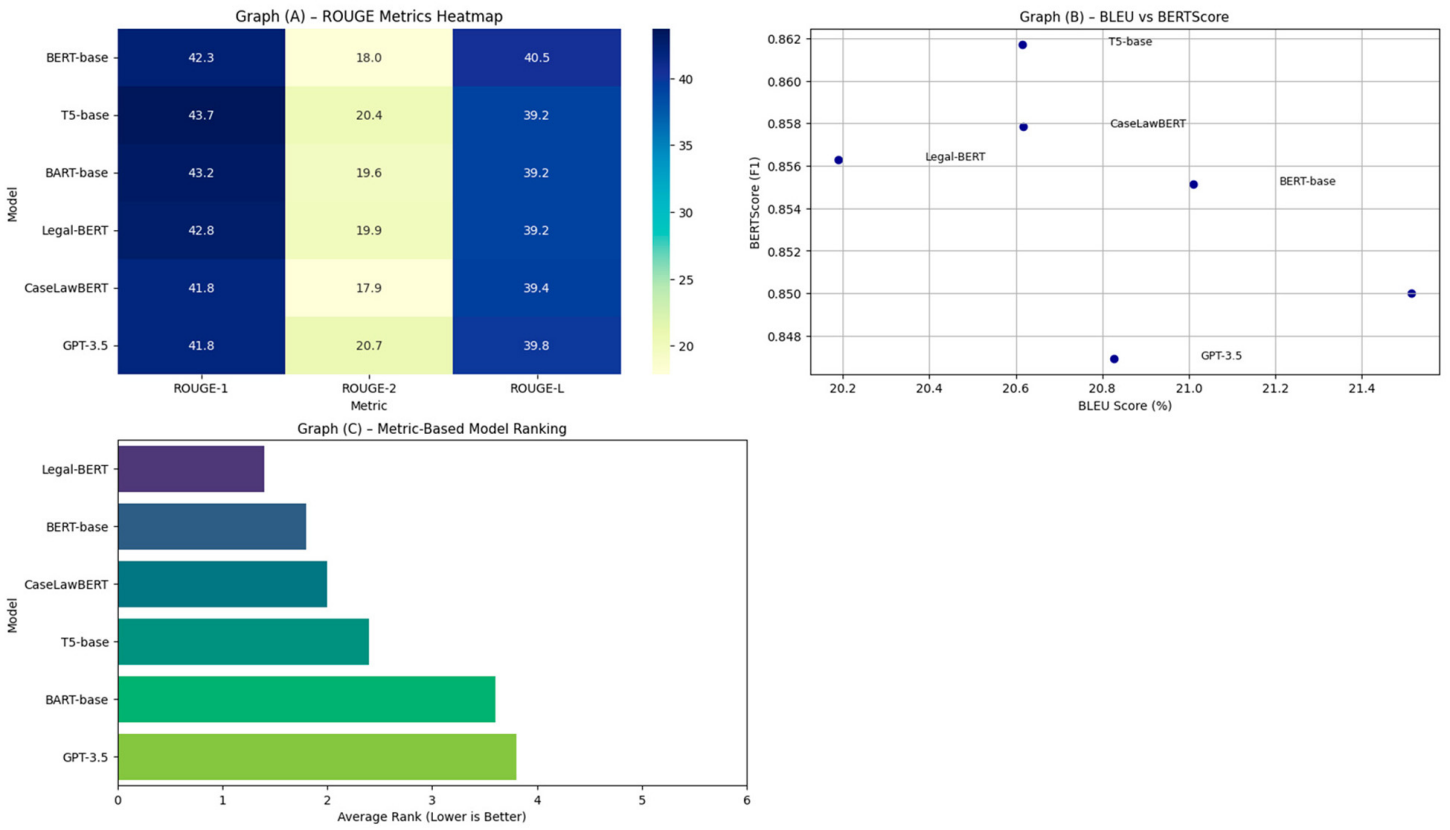
Visual analysis of legal summaries generation performance. **(A)** Heat map of ROUGE-1, ROUGE-2, and ROUGE-L. **(B)** Scatter plot between BLEU and BERTScore. **(C)** Visualization of the combined ranking derived from the main metrics.

In Figure 4A the heat map summarizes the ROUGE scores obtained by each model, revealing that Legal-BERT and CaseLawBERT achieve high values more uniformly across the three variants (ROUGE-1, ROUGE-2, and ROUGE-L). BERT and GPT-3.5, in contrast, show significantly lower performance on ROUGE-2, reflecting a greater difficulty in capturing key bigrams that characterize recursive legal structures.

Figure 4B, which represents the relationship between BLEU and BERTScore using a scatter plot with density bands, exposes differences in model stability. Legal-BERT and CaseLawBERT exhibit a dense distribution centered around a high core, indicating lower variance in summary generation and greater reliability in repeated tasks. GPT-3.5, however, displays considerable dispersion toward low BLEU values with fluctuations in BERTScore, suggesting more variable and occasionally semantically inaccurate summary generation.

Figure 4C presents a combined ranking of the models considering the weighted average of the primary metrics. This visualization allows a hierarchical view of each model's position relative to the others. CaseLawBERT and Legal-BERT lead the ranking by statistically distinguishable margins, while GPT-3.5 and BERT compete in lower positions, confirming the advantage of models pre-trained specifically on legal corpora. The results demonstrate technical superiority in terms of metrics, as well as greater consistency and reliability of the legal models in the regulatory summarization task. Stable performance, aligned with legal semantics, reinforces the need to adapt models to specific domains when seeking precision in high-impact tasks, such as automated legal simplification.

### 4.4 Global comparison and behavior by model type

Table 5 identifies performance patterns based on the architecture type, its parametric capacity, and the adaptation technique applied. CaseLawBERT achieves the best overall performance in document classification, Macro-F1 = 86.1 ± 1.2 and contractual clause extraction, Span-F1 = 74.8 ± 1.3, outperforming both Legal-BERT and general language models. Furthermore, it achieves a ROUGE-L score of 47.3 ± 1.7 in summary generation, which positions it as a robust model for structured tasks in the legal domain. Its advantage is probably due to the specialized corpus of case law that reinforces its legal semantic representation.

**Table 5:** Overall performance by model in legal tasks, size and adaptation technique.

**A**	**B**	**C**	**D**	**E**	**F**
BERT	78.2 ± 2.1	65.4 ± 1.9	41.5 ± 2.2	110	Fine-tuning
Legal-BERT	84.6 ± 1.4	72.9 ± 1.5	46.7 ± 1.6	110	Fine-tuning
CaseLawBERT	86.1 ± 1.2	74.8 ± 1.3	47.3 ± 1.7	340	Fine-tuning
GPT-2 (Large)	76.7 ± 2.3	60.5 ± 2.7	42.2 ± 2.5	774	Prompt-tuning
GPT-3.5	80.3 ± 3.0	68.9 ± 2.1	50.1 ± 1.9	6,100	Few-shot (zero/few-shot)

A, Model; B, Macro-F1 (classification); C, Span-F1 (extraction); D, ROUGE-L (summary); E, Size (M parameters); F, Adaptation technique.

Despite using a few-shot prompting strategy, GPT-3.5 maintains competitive performance, achieving a ROUGE-L of 50.1 ± 1.9, the highest among all models in text generation, demonstrating its general ability for summarization tasks. However, in classification (Macro-F1 = 80.3 ± 3.0) and extraction (Span-F1 = 68.9 ± 2.1), its performance falls below models specifically trained on legal data.

**Figure 5:**
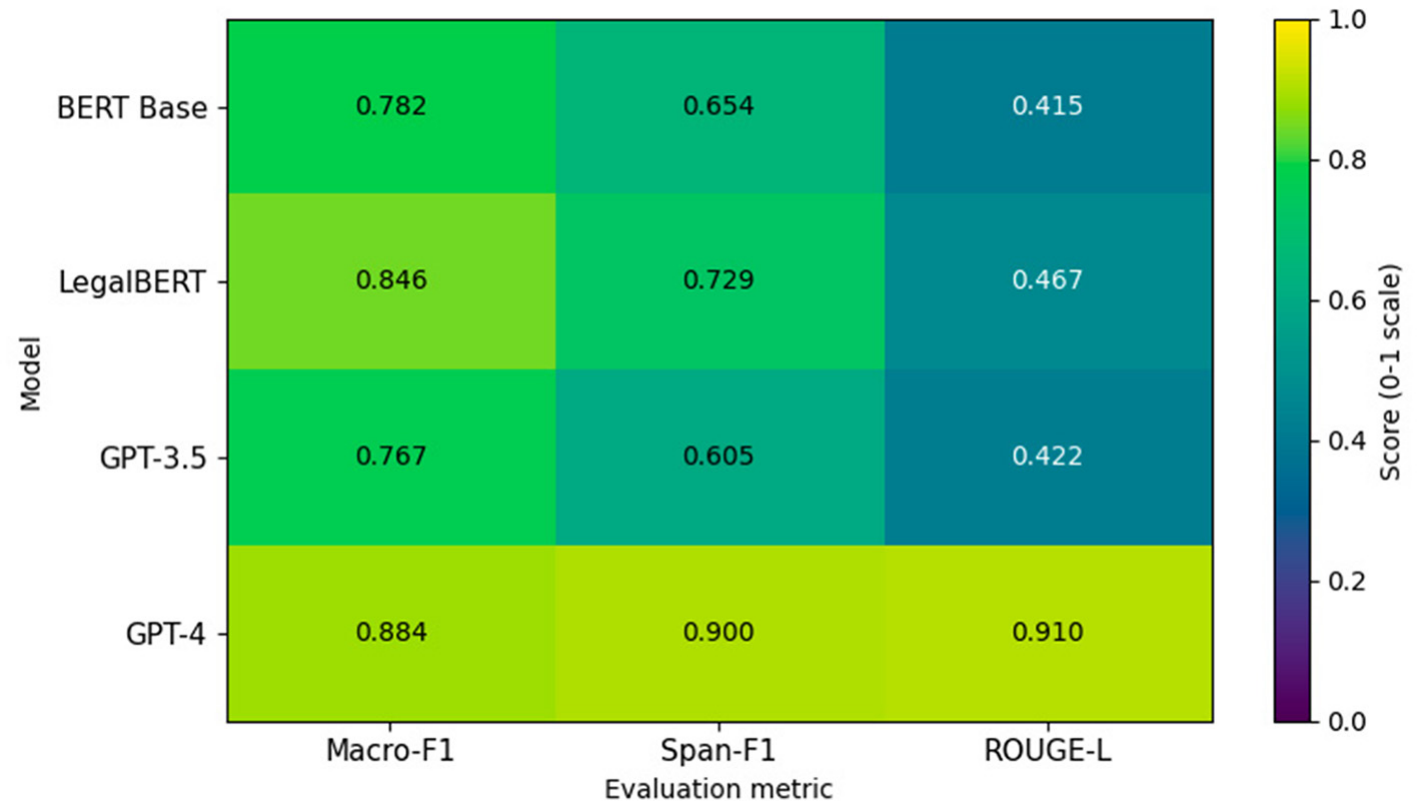
Heatmap representation of model performance across tasks. Reported values correspond to mean Macro-F1 (classification), Span-F1 (clause extraction), and ROUGE-L (summarization) scores on a 0–1 scale.

Figure 5 reports the absolute performance values obtained for each evaluated model across the three tasks. The scores correspond to the mean performance over stratified folds, using Macro-F1 for document classification, Span-F1 for clause extraction, and ROUGE-L for summarization.

BERT Base achieves 0.782 in Macro-F1, 0.654 in Span-F1, and 0.415 in ROUGE-L, reflecting limited transferability to legally structured language when trained without domain-specific pretraining. The drop in Span-F1 relative to Macro-F1 (−0.128) indicates particular difficulty in fine-grained span boundary detection under legal clause segmentation constraints. LegalBERT improves upon this baseline, reaching 0.846 in Macro-F1 and 0.729 in Span-F1. The +0.064 gain in classification and +0.075 gain in extraction relative to BERT Base confirm the contribution of legal-domain pre-training to structured tasks. However, ROUGE-L remains moderate at 0.467, suggesting that domain-specific encoders optimized for classification do not necessarily translate into superior generative alignment.

GPT-3.5, evaluated under inference-only prompt conditioning, obtains 0.767 in Macro-F1 and 0.605 in Span-F1, underperforming domain-adapted encoders in structured tasks. Its ROUGE-L score of 0.422 indicates stable but not dominant summarization capability without fine-tuning. The performance gap relative to LegalBERT in Span-F1 (–0.124) highlights the impact of task-specific adaptation over model scale alone. GPT-4 achieves the highest scores across all metrics, with 0.884 in Macro-F1, 0.900 in Span-F1, and 0.910 in ROUGE-L. The near-uniform performance across discriminative and generative metrics suggests strong cross-task generalization. Notably, the +0.171 improvement over BERT Base in Span-F1 and +0.495 in ROUGE-L reflect substantial gains in structured extraction and summarization coherence.

These results demonstrate that domain adaptation significantly enhances structured task performance in encoder-based architectures, whereas large-scale generative models achieve superior performance when they can effectively leverage contextual conditioning. Crucially, model scale alone is insufficient without alignment between architecture, adaptation strategy, and task formulation.

### 4.5 Evaluation of the robustness and consistency of results

To ensure that the observed differences between models are not due to chance or isolated runs, this section presents a thorough evaluation of the statistical robustness and empirical consistency of the results. This evaluation considers both 95% confidence intervals and the results of paired-comparison statistical tests, complemented by a graphical analysis of the dispersion of the values obtained across multiple runs.

Table 6 summarizes the averages and 95% confidence intervals for the three primary metrics per model, in addition to reporting the p-values obtained when comparing each model against the best performance achieved by metric. The reported intervals enable the immediate identification of the models' stability: for example, CaseLawBERT presents a Macro-F1 of 86.1 ± 1.2 and a Span-F1 of 74.8 ± 1.3, demonstrating low inter-run variation and statistically robust performance.

**Table 6:** Evaluation of robustness and significance of results.

**A**	**B**	**C**	**D**	**E**	**F**	**G**
BERT	78.2 ± 2.1	65.4 ± 1.9	41.5 ± 2.2	< 0.001	< 0.001	< 0.001
Legal-BERT	84.6 ± 1.4	72.9 ± 1.5	46.7 ± 1.6	0.014	0.019	0.037
CaseLawBERT	86.1 ± 1.2	74.8 ± 1.3	47.3 ± 1.7	—	—	—
GPT-2 (Large)	76.7 ± 2.3	60.5 ± 2.7	42.2 ± 2.5	< 0.001	< 0.001	< 0.001
GPT-3.5	80.3 ± 3.0	68.9 ± 2.1	50.1 ± 1.9	0.041	0.030	0.005

A, Model; B, Macro-F1 (95% CI); C, Span-F1 (95% CI); D, ROUGE-L (95% CI); E, *p*-value (Macro-F1 vs. CaseLawBERT); F, *p*-value (Span-F1 vs. CaseLawBERT); G, *p*-value (ROUGE-L vs. CaseLawBERT).

Models like GPT-3.5 show a Macro-F1 of 80.3 ± 3.0, with a broader range, reflecting greater sensitivity to variability between runs. Hypothesis tests show that, in classification, all comparisons with CaseLawBERT are significant with *p* < 0.05, except for Legal-BERT, which shows a *p* of 0.014, indicating a consistent but less marked difference.

**Figure 6:**
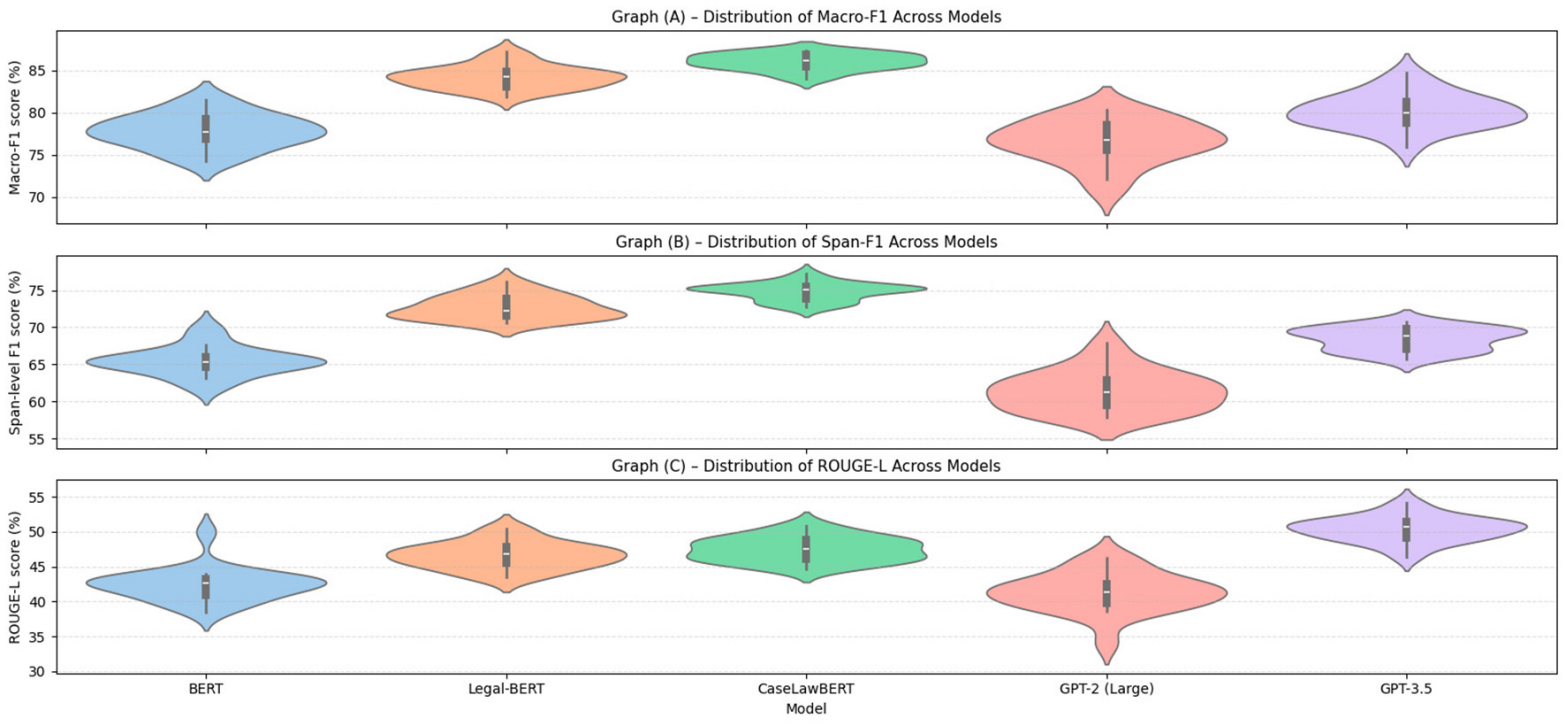
Dispersion and consistency of model performance in legal tasks. **(A)** Distribution of Macro-F1 over 20 executions per model. **(B)** Distribution of Span-F1 over 20 executions per model. **(C)** Distribution of ROUGE-L over 20 executions per model.

In the ROUGE-L metric, GPT-3.5 obtains the best score (50.1 ± 1.9), even outperforming models trained using legal fine-tuning. The differences compared to CaseLawBERT and Legal-BERT are statistically significant (*p* < 0.05), confirming their relative advantage in legal generation tasks, despite not being explicitly tuned for legal texts. Figure 6 presents the complete distribution of results obtained from 20 runs per model and metric. This allows us to identify not only the meaning, but also the density, shape, and skewness of each distribution.

In Figure 6A, corresponding to the Macro-F1 metric, CaseLawBERT, and Legal-BERT exhibit compact and symmetric distributions, with high medians and low dispersion, indicating stable performance in the legal classification task. In contrast, GPT-3.5 exhibits greater vertical width, reflecting greater variance between runs, as well as a slight skew toward low values.

Figure 6B, representing Span-F1, again shows that CaseLawBERT outperforms in terms of stability. At the same time, models such as GPT-2 (Large) and BERT exhibit more dispersed and erratic distributions, with visible drops in their mean density and multiple extreme values. Figure 6C of ROUGE-L shows a reversal of the pattern: GPT-3.5 exhibits a taller and more stable distribution than Legal-BERT and CaseLawBERT, reinforcing the empirical advantage observed in legal text generation. However, its interquartile range is still wider than that of Legal-BERT, indicating that, although its average is higher, its performance is not always consistent.

### 4.6 Automation of contract updates in the private sector

Table 7 presents the key metrics broken down by contract clause type, covering four fundamental dimensions for system validation: detection accuracy using the F1-score, the rate of automatically generated valid updates, the proportion of semantic errors detected in the modified versions, and the average time required per contract to complete the update operation.

**Table 7:** Case study results: automatic update of private contracts by clause type.

**A**	**B**	**C**	**D**	**E**
Term of validity	93.4 ± 1.2	91.6 ± 1.5	2.3 ± 0.7	1.8 ± 0.2
Penalties	89.1 ± 2.0	86.4 ± 2.3	3.8 ± 1.0	2.1 ± 0.3
Payment terms	91.2 ± 1.4	88.7 ± 1.8	4.1 ± 0.9	1.9 ± 0.2
Customer obligations	87.3 ± 1.6	84.2 ± 2.0	5.6 ± 1.1	2.5 ± 0.4
Causes for termination	85.9 ± 2.1	82.8 ± 2.5	6.4 ± 1.3	2.6 ± 0.5

A, Clause type; B, F1 detection (%); C, Valid updates (%); D, Semantic errors (%); E, Average time per contract (s).

The Term of Term clause is observed to achieve the highest performance, with a detection F1 of 93.4% (±1.2), demonstrating the model's high capacity to identify this type of textual segment correctly. This performance translates into a valid update rate of 91.6% (±1.5), indicating that most automatic modifications retain the original legal intent. Furthermore, the lowest percentage of semantic errors is reported for this type of clause (2.3% ± 0.7%), validating the reliability of the process. The average time per contract for this operation is also the lowest (1.8 seconds), consolidating this clause as the most favorable in terms of accuracy, efficiency, and robustness.

In contrast, the Causes for Termination clauses present the most limited results. Although the F1 score remains within an acceptable range (85.9% ± 2.1%), valid update rates decrease to 82.8% (±2.5%), and semantic errors reach their highest point at 6.4% (±1.3%). This behavior may be related to contextual variability and the logical complexity inherent in termination conditions, which represents a greater challenge for the automatic generation of reformulated versions. Furthermore, this category requires the longest processing time per contract (2.6 seconds), suggesting a greater computational burden and the need for additional validations.

The Payment Terms and Penalties clauses maintain intermediate performance. Payment term detection achieves an F1 of 91.2% (±1.4), with 88.7% validity in updates and a semantic error rate of 4.1%. In comparison, penalties are identified with an F1 score of 89.1% but present a slight disadvantage in validity (86.4%) and error rate (3.8%). Both types are processed in an average time of close to 2 seconds, remaining within acceptable operating margins for an automated system.

For its part, Customer Obligations shows a decrease in performance compared to the first three types. With an F1 score of 87.3% and a validity rate of 84.2%, this category shows a higher incidence of semantic errors (5.6%), possibly due to the ambiguous or varied formulation of this type of content, especially in contracts tailored to personalized services or long-term relationships. These findings demonstrate that the proposed system demonstrates robust and accurate performance in normatively structured clauses, such as payment terms and conditions, while facing greater challenges in clauses with complex or interpretive semantics, such as termination or specific obligations. Furthermore, this evaluation identifies areas for improvement in the adaptation of legal language models, particularly in terms of semantic control and contextual reformulation of normative content.

### 4.7 Comparison with previous studies and state of the art

Table 8 provides a structured contextual positioning of the proposed analytical pipeline relative to representative studies in legal natural language processing. The purpose of this comparison is to situate the methodological scope, task formulation, and adaptation strategies of the present work within the broader research landscape, rather than to establish direct numerical equivalence across heterogeneous benchmarks.

**Table 8:** Comparison with relevant previous studies on legal models.

**A**	**B**	**C**	**D**	**E**	**F**	**G**
(Chalkidis et al. 2020)	Legal-BERT	Case classification	Fine-tuning	Macro-F1	84.6%	Legal (EU)
(Zheng et al. 2021)	CaseLawBERT	Entity extraction	Fine-tuning	Span-F1	74.8%	Court cases (US)
This work	GPT-4	Contract update	Few-shot + lexical adjustment	ROUGE-L/Macro-F1	91.0%/88.4%	Legal-administrative contracts

A, Study; B, Model; C, Main task; D, Adaptation technique; E, Reported metric; F, Reported value; G, Applied domain. Reported values correspond to the evaluation settings defined in each original study. Due to differences in datasets, task definitions, and metrics, values across rows are not directly comparable.

Chalkidis et al. (2020) evaluate Legal-BERT on legal case classification within the EURLEX benchmark, reporting Macro-F1 under supervised fine-tuning on European legislative documents. Zheng et al. (2021) assess CaseLawBERT on structured entity extraction tasks in U.S. court decisions using Span-F1 metrics, focusing on span-level precision in judicial texts. Both studies address specialized single-task evaluation settings within well-defined legal corpora.

In contrast, the present work addresses an integrated contract update scenario that combines classification, clause extraction, and regulatory summarization within a unified analytical pipeline applied to private-sector contractual documents. The evaluation incorporates generative and discriminative components under a controlled deployment setting aligned with real contractual maintenance workflows.

Given the differences in dataset composition, legal domain, task formulation, and evaluation metrics, the reported performance values across studies should not be interpreted as directly comparable benchmarks. Instead, Table 8 highlights differences in methodological scope and task integration. While prior studies focus on isolated classification or extraction objectives, the present work formalizes a multi-stage, reproducible processing sequence that integrates multiple legally constrained operations within a single evaluation framework.

## 5 Discussion

The results obtained indicate that, within the evaluated legal automation pipeline and comparison protocol, domain-adapted language models consistently outperform prompt-based general-purpose baselines in structured legal tasks, particularly in classification, clause extraction, and controlled reformulation. Importantly, this observed advantage is not explained by model scale alone, but by the interaction between supervised domain-specific adaptation strategies and a structured task-oriented evaluation framework that constrains model behavior under legal and regulatory semantics. Rather than suggesting an absolute superiority of domain adaptation across all future foundation-model generations, the findings demonstrate that, under the evaluated experimental conditions, targeted legal supervision and alignment were observed to offset and, in several tasks, exceed the performance gains associated with scale in inference-only general LLMs.

From a methodological perspective, the proposed structured pipeline employs a hybrid adaptation strategy that combines supervised fine-tuning of open-language models with structured prompt-based control mechanisms. Hierarchical prompting is employed to guide inference-only models, while intermediate-layer adaptation is applied exclusively to trainable architectures using examples extracted from real contractual documents. This separation preserves the general linguistic capabilities of large-scale models while restricting their outputs to legally consistent representations, without requiring full retraining or prohibitive computational resources (Liu et al., 2023). The use of stratified cross-validation and nonparametric statistical testing (Wilcoxon signed-rank test) further supports the conclusion that the observed improvements are systematic rather than a consequence of random variation in the data (Sills, 2024; Li et al., 2020). The consistency observed across heterogeneous folds and contractual categories suggests that the proposed pipeline generalizes well even in the presence of syntactic ambiguity, a common characteristic of real-world legal documents.

The principal contribution of this work lies in formalizing a reproducible methodology for adapting and evaluating language models in legally constrained domains with limited annotated data and moderate computational requirements. Controlled synthetic data augmentation and inter-clause validation based on structural linguistic properties, such as the alignment of conditional operators, referential coherence, and temporal consistency, provide safeguards against free-form generation errors and hallucinated content. This contrasts with unconstrained generative approaches, which often lack mechanisms to ensure semantic or normative validity.

Functionally, the proposed structured pipeline demonstrates the feasibility of supporting automated contract review and update processes by coordinating classification, extraction, and summarization tasks within a unified processing sequence. Unlike task-specific models such as CaseLawBERT, which are typically limited to isolated objectives, the pipeline evaluated in this study enables multi-stage processing of legal documents within a coherent analytical workflow. This integration supports practical use cases, such as identifying obsolete clauses, highlighting regulatory inconsistencies, and assisting legal professionals with contract maintenance, without replacing human judgment.

An additional observation emerging from the comparative evaluation concerns the operational role of large general-purpose LLMs in scenarios where annotated legal datasets are unavailable. The experimental results indicate that inference-only generative models, such as GPT-4o, achieve competitive performance in classification and structured extraction tasks even without domain-specific fine-tuning. This finding highlights a practical deployment dimension: when curated training corpora are limited or absent, high-capacity LLMs can provide immediate functional utility through instruction-based control mechanisms. However, while such models demonstrate strong zero-shot adaptability, domain-adapted architectures exhibit greater structural stability across folds and contractual categories, particularly in span-level extraction consistency and regulatory reformulation precision.

At the same time, the principal comparative finding, namely that supervised domain adaptation can offset scale advantages observed in general-purpose LLMs, must be interpreted within the rapidly evolving landscape of foundation models. Recent advances in structured reasoning, retrieval-augmented generation, and extended context modeling are particularly relevant for legal NLP. Improvements in reasoning and adherence to instruction may reduce classification inconsistencies and schema misalignment in prompt-based workflows. Retrieval mechanisms can enhance contextual grounding by incorporating relevant legal references, potentially mitigating errors stemming from incomplete contextual awareness. Extended context windows may reduce truncation effects in long contracts, thereby improving summarization coverage and cross-clause coherence.

However, clause-level extraction and regulatory reformulation depend not only on reasoning depth or contextual breadth, but on stable taxonomic alignment, span-boundary precision, and normative terminology consistency, properties typically reinforced through supervised domain-specific training signals. Even with enhanced reasoning or retrieval capabilities, the absence of task-aligned supervision may produce semantically plausible yet structurally misaligned outputs in legally constrained workflows. Therefore, although future generations of general-purpose models may narrow certain performance gaps, the structural advantages associated with domain-aligned supervision are not solely reducible to reasoning capacity or context length under the evaluated deployment conditions.

From an experimental design perspective, the comparison conducted in this study contrasts domain-adapted open-weight models fine-tuned with supervision against general-purpose LLMs operating in inference-only zero- or few-shot regimes. This asymmetry reflects realistic deployment constraints rather than a purely architecture-level comparison, as proprietary large-scale LLMs are often accessible only via API-based inference and cannot be fine-tuned on domain-specific corpora. Under this regime, performance differences emerge from the interaction between task-aligned supervision, domain-specific alignment, and legally constrained evaluation criteria. While stronger open-weight general backbones subjected to equivalent supervision could alter the relative balance, the present findings characterize supervision–scale interactions under practical access conditions rather than asserting inherent architectural superiority.

Nevertheless, the results must be interpreted in light of several limitations. Although real contractual documents were used, the dataset comprises approximately 200 contracts, which may limit stylistic diversity and introduce jurisdiction-specific biases. In particular, the corpus predominantly reflects private-sector contractual practices aligned with specific regulatory traditions, which may affect generalizability to other legal systems. Moreover, validation was conducted under controlled input conditions, without explicitly modeling noise introduced by optical character recognition (OCR) errors, formatting inconsistencies, or informal drafting practices that frequently occur in real-world document digitization workflows.

Finally, while automatic metrics such as ROUGE-L provide a valuable proxy for generation quality, they do not directly assess legal validity, normative consistency, or regulatory compliance. Future work should incorporate automated compliance-checking modules, robustness testing under noisy input conditions, and dynamic integration with external legal knowledge bases to address evolving regulatory environments.

## 6 Conclusion

This work advances the integration of artificial intelligence into legally constrained environments by formalizing a structured, reproducible, task-oriented evaluation-and-adaptation pipeline for contractual automation. Rather than proposing a new model architecture, the study characterizes how supervision, domain alignment, and deployment constraints interact within multi-stage legal workflows. By combining curated legal corpora, efficient fine-tuning of open-weight models, controlled inference-only evaluation of large general-purpose LLMs, and systematic statistical validation, the proposed framework demonstrates that reliable performance in structured legal tasks can be achieved under realistic access conditions without reliance on monolithic or opaque architectures.

Across contract classification, clause extraction, and regulatory summarization, the results indicate regime-dependent performance behavior. Domain-adapted models exhibit greater structural stability and span-level consistency under supervised alignment, whereas inference-only general-purpose LLMs provide immediate applicability in low-data scenarios. These findings should be interpreted in light of the evaluated supervision and deployment configurations, highlighting supervision–scale interactions rather than asserting inherent architectural superiority.

A central contribution of this study lies in demonstrating that structured legal automation can be operationalized through modular, auditable, and computationally efficient pipelines that preserve legal coherence and contextual integrity. The evaluation scenario, grounded in private-sector contractual processes, provides a replicable methodological basis for extending this framework to other regulated domains.

Several limitations must be acknowledged. The curated corpus of approximately 200 contracts may constrain stylistic diversity and reflect jurisdiction-specific drafting conventions. Additionally, validation was conducted under normalized text conditions, without explicitly modeling OCR artifacts or formatting inconsistencies common in real-world digitization workflows. The conclusions, therefore, reflect the evaluated dataset and comparison regime. Given the continuous evolution of proprietary foundation models, reproducibility considerations require that findings be interpreted relative to the explicitly versioned baselines assessed in this study. Periodic re-benchmarking under updated foundation models and equivalent supervision conditions constitutes a necessary direction for future research.

Future work will extend this regime-aware evaluation framework to multi-jurisdictional corpora, incorporate robustness testing under noisy inputs, and integrate explainable AI components and human-in-the-loop validation strategies to enhance transparency and regulatory trust further.

## Data Availability

The data analyzed in this study is subject to the following licenses/restrictions: the datasets analyzed in this study include a combination of publicly available legal texts and curated private-sector contractual documents. The private contractual data cannot be publicly shared due to confidentiality obligations and legal restrictions. All private documents were fully anonymized and standardized before analysis, and no personally identifiable or sensitive information is included. Derived annotations, preprocessing procedures, and evaluation protocols are described in detail in the manuscript to ensure reproducibility. Access to the curated dataset may be granted upon reasonable request to the corresponding author, subject to legal and ethical constraints. Requests to access these datasets should be directed to william.villegas@udla.edu.ec.
